# Diagnostic delay in rare diseases in the Campania region: addressing ageing, gender disparities, and the “postcode lottery effect” to reduce the patient odyssey

**DOI:** 10.1093/eurpub/ckaf088

**Published:** 2025-07-04

**Authors:** Chiara Cirillo, Rossella Duraccio, Mario Fordellone, Emanuele Monda, Bruno De Rosa, Chiara De Stasio, Anna Francesca Smimmo, Anna Fusco, Salvatore Rega, Martina Caiazza, Francesca Marzullo, Raffaele Scarpa, Gioacchino Scarano, Antonio Postiglione, Pietro Buono, Ugo Trama, Massimo Di Gennaro, Giuseppe Borriello, Maria Galdo, Barbara Morgillo, Paolo Chiodini, Giuseppe Limongelli

**Affiliations:** Inherited and Rare Cardiovascular Diseases, Department of Translational Medical Sciences, University of Campania “Luigi Vanvitelli”, Via Leonardo Bianchi, Naples, 80131, Italy; Department of Mental, Physical Health and Preventive Medicine, University of Campania “Luigi Vanvitelli”, Naples, Italy; Department of Mental, Physical Health and Preventive Medicine, University of Campania “Luigi Vanvitelli”, Naples, Italy; Inherited and Rare Cardiovascular Diseases, Department of Translational Medical Sciences, University of Campania “Luigi Vanvitelli”, Via Leonardo Bianchi, Naples, 80131, Italy; Department of Mental, Physical Health and Preventive Medicine, University of Campania “Luigi Vanvitelli”, Naples, Italy; Centro di Coordinamento Malattie Rare, Regione Campania, Naples, Italy; Centro di Coordinamento Malattie Rare, Regione Campania, Naples, Italy; Department of Mental, Physical Health and Preventive Medicine, University of Campania “Luigi Vanvitelli”, Naples, Italy; Centro di Coordinamento Malattie Rare, Regione Campania, Naples, Italy; Centro di Coordinamento Malattie Rare, Regione Campania, Naples, Italy; Inherited and Rare Cardiovascular Diseases, Department of Translational Medical Sciences, University of Campania “Luigi Vanvitelli”, Via Leonardo Bianchi, Naples, 80131, Italy; Centro di Coordinamento Malattie Rare, Regione Campania, Naples, Italy; Centro di Coordinamento Malattie Rare, Regione Campania, Naples, Italy; Inherited and Rare Cardiovascular Diseases, Department of Translational Medical Sciences, University of Campania “Luigi Vanvitelli”, Via Leonardo Bianchi, Naples, 80131, Italy; General Directorate of Health, Campania Region, Naples, Italy; General Directorate of Health, Campania Region, Naples, Italy; General Directorate of Health, Campania Region, Naples, Italy; Soresa Direzione Innovazione e Sanita’ Digitale, Naples, Italy; Soresa Direzione Innovazione e Sanita’ Digitale, Naples, Italy; Department of Pharmacy, AORN Ospedali dei Colli-Monaldi Hospital, Naples, Italy; General Directorate of Health, Campania Region, Naples, Italy; Department of Mental, Physical Health and Preventive Medicine, University of Campania “Luigi Vanvitelli”, Naples, Italy; Inherited and Rare Cardiovascular Diseases, Department of Translational Medical Sciences, University of Campania “Luigi Vanvitelli”, Via Leonardo Bianchi, Naples, 80131, Italy

## Abstract

Our study assessed the time to diagnosis of rare diseases (RDs) in Campania and whether there are determinants of diagnostic delay (DD). Demographic characteristics, date of first medical contact and diagnosis, disease macro-groups, and area of residence of patients were recorded. DD was calculated as the time elapsed (in years) from the onset of symptoms to the RD diagnosis date. Based on the Rare Disease Research Consortium consensus document, a time to diagnosis more than one year was considered DD. A multilevel logistic regression was performed. Seven thousand nine hundred and nine patients were included in the analysis; 47.4% were male. The mean DD was 3.4 years and 46% of patients experienced DD. Predictors of DD were female gender (OR 0.90, 95% CI 0.80–0.98, *P* < .005), age at diagnosis (OR 1.36, 95% CI 1.27–1.45, *P* < .001), and province of residence (residence in Naples vs. others; OR 0.80, 95% CI 0.73–0.88, *P* < .001). Immunological, connective tissue, digestive, genitourinary system diseases, and congenital malformations showed more DD than other disorders. Nearly half of the patients with RD experienced DD. The main determinants of DD were female sex, older age at diagnosis.

## Introduction

A disease is considered rare when it affects no more than one person in 2000. While each rare disease (RD) has a low incidence, collectively, RDs affect 3.8% to 5% of the population [1].

The International Rare Disease Research Consortium (IRDiRC) was established to address challenges in RD diagnosis and management through research, aiming to diagnose patients with RD within one year of their initial medical consultation, provided that the disorder is documented in the medical literature [2].

In Italy, to be exempt from medical costs related to an RD and to access specific therapies, one must obtain an RD certification of diagnosis from a recognized national or regional expert on the specific disease. Since 2001, Italy has implemented a structured system to provide healthcare to RD patients, based on a government ruling that promotes the development of regional Rare Disease Registries (RDR), managed by RD Regional Coordinator Centres (RDRCC), linked to the National Registry of Rare Diseases [3], managed by the RD National Coordinator Centres (RDNCC) at Istituto Superiore di Sanità (ISS).

Patients with RD often face significant diagnostic delay (DD) due to limited local knowledge of the disease, delays in accessing specialists, nonspecific symptoms, and generally challenging diagnoses. DD affects not only the quality of life for rare disease patients but also their access to new therapies and broader support.

A recent Europe-wide RD patient survey reported an average time to diagnosis of nearly 5 years, and a retrospective analysis of the Spanish RD registry found an average DD of 6.8 years. Both studies identified female gender as a key determinant of DD; regarding age groups at higher risk, the Spanish data indicated that those aged 30–44 faced the greatest delay, while the European survey found higher DD in those under 30 [4, 5].

Our study aimed to assess time to diagnosis of RD in Campania Region (Southern Italy, Supplementary Fig. S1 illustrates demographics) and to identify whether certain patient subsets are at higher risk of significant DD.

## Methods

### Study design

A retrospective observational registry analysis was conducted, adhering to the principles of the Declaration of Helsinki and its amendments, and approved by the Ethical Committee (protocol n AOC-0013766-2020; 08/05/2020).

The Campania Region RD registry was retrospectively screened, and all patients who received a certification of RD in Campania region between 1 January 2018 and 31 December 2022 were included in the analysis. Anonymized clinical data, including non-identifiable demographic characteristics, RD macro-group, date of first medical contact for symptoms, date of diagnosis, and province of residence, were collected. Cases missing any of this information were excluded. RD was grouped in 15 macro-groups with groups 4 and 15 further divided into subgroups as follows:

1. Infectious disease;
2. Tumors;
3. Endocrine disorders;
4. Metabolic disorders:
  - Congenital defects of mitochondrial energy metabolism;
  - Lysosomal storage disorders;
  - Congenital defects of the absorption and transport of vitamins;
  - Congenital defects of the absorption and transport of proteins;
  - Congenital defect of the metabolism and transport of metals;
5. Immunological disorders;
6. Blood and hematopoietic organs disorders;
7. Nervous system disorders;
8. Ophthalmological disorders;
9. Circulatory system disorders;
10. Respiratory system disorders;
11. Digestive system disorders;
12. Genitourinary system disorders;
13. Dermatological disorders;
14. Connective tissue and musculoskeletal systems disorders;
15. Congenital malformations (inherited and genetic):
  - Congenital malformative syndromes with predominant alteration of the nervous system;
  - Congenital malformations of the heart, great vessels, and peripheral vessels;
  - Congenital malformations of the digestive system isolated and syndromic;
  - Genetic disease of the skeletal system;
  - Other syndromes and complex congenital malformations.

If the diagnosis was made before birth, the birthdate was assumed as both the date of symptom onset and the date of diagnosis. Clinically significant DD was defined as a time to diagnosis of more than one year, as per IRDiRC recommendations, and severe DD was defined as a time to diagnosis of over four years [2].

### Statistical analysis

Continuous variables were reported as means and standard deviations or medians and interquartile ranges (IQRs) based on their distribution, as assessed by the Shapiro–Wilk normality test. Categorical variables were reported as percentages. DD was calculated as the time elapsed (in years) from the date of symptom onset to the date of RD diagnosis.

To analyse DD among different groups a multilevel logistic regression model was performed. The best model in terms of fitting was selected with Akaike’s Information Criterion. The best model included sex at birth, age of diagnosis, year of diagnosis, and residence in Naples as fixed effects. Random intercepts for disease macro-groups were used, which allowed for the definition of the variation of DD from the average, and their statistical significance has been assessed in comparison to 95% confidence intervals (CIs). All results are presented as odds ratio (OR), 95% CI, and two-tailed *P*-values. Interdependence among statistical units belonging to the same diseases macro-group has been evaluated using an interclass correlation coefficient (ICC). Statistical tests with *P*-values <.05 considered statistically significant. All the statistical analyses were performed with the R Studio Statistical software, version 4.1.3.

## Results

**Figure 1.**
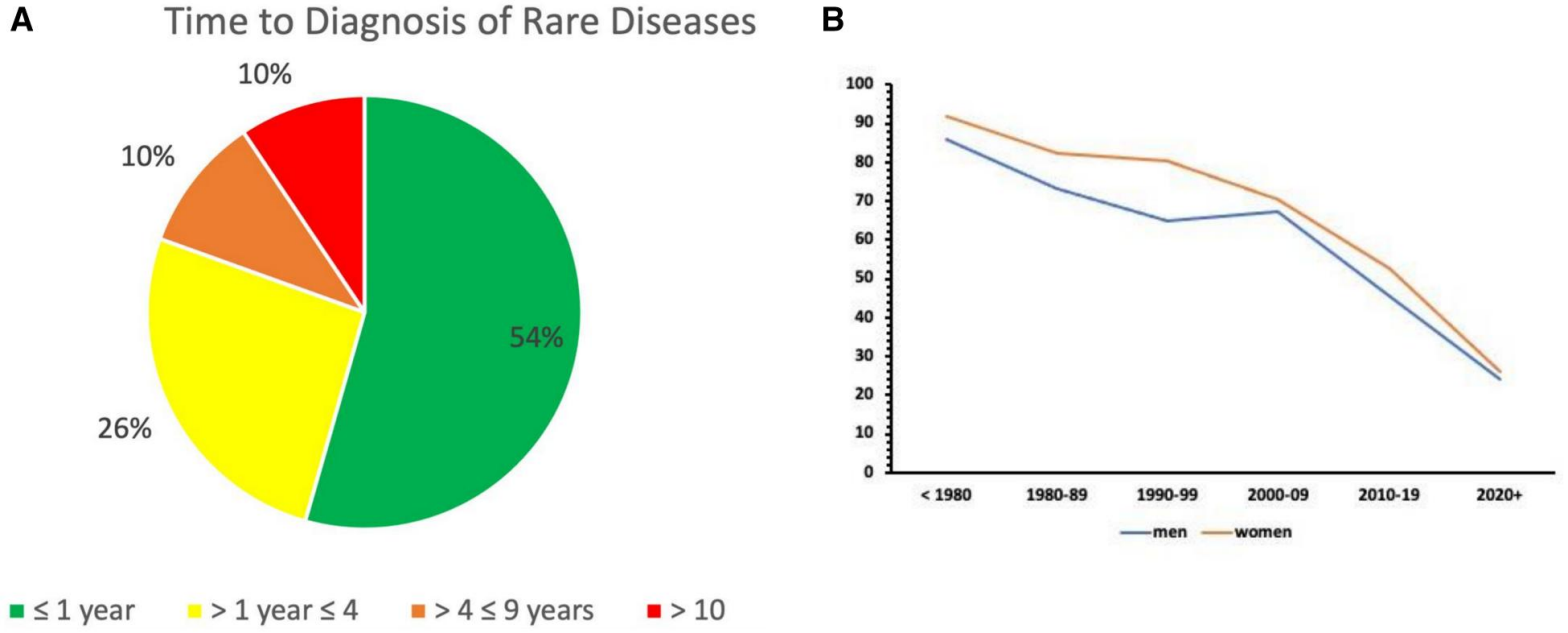
(A) Time to diagnosis of rare disease. (B) Prevalence of diagnostic delay for rare diseases over time by gender.

A total of 7909 patients were included in the analysis, of whom 47.4% were male. The mean age of disease onset was 32.9 (±25.8) years, and the median was 31.81. The average time to RD diagnosis in Campania was 3.4 (±7.2) years. Forty-six percent of patients experienced a DD of over 1 year (Table 1). Time to diagnosis was less than 1 year in 54% of patients, between 1 and 4 years in 26%, between 4 and 9 years in 10%, and over 10 years in 10% (Fig. 1A). The prevalence of DD decreased over the years, as shown in Fig. 1B.

**Table 1.** Baseline characteristics

*N*	7909
Male	47.4% (3749)
Age at symptom onset	32.9 (7.1–58.7)
Age at diagnosis	36.3 (10.9–61.7)
Time to diagnosis, mean	3.4 (±7.2)
Time to diagnosis >1 year	45.6%
Resident in Naples	54.1%

Results from the multilevel logistic regression model showed that male sex (OR 0.90, 95% CI 0.80–0.98, *P* < .005) and residence in Naples (OR 0.80, 95% CI 0.73–0.88, *P* < .001) were associated with a statistically significant lower DD, while older age at diagnosis (OR 1.36, 95% CI 1.27–1.45, *P* < .001) was associated with a significantly higher DD. The intraclass correlation coefficient (ICC) was 0.22, indicating poor reliability (Table 2).

**Table 2.** Estimated regression coefficients and variance components in the multilevel logistic regression model

Independent variables		
	OR (95% CI)	*P*-value
Gender: male	0.90 (0.80–0.98)	<.05
Age at diagnosis	1.36 (1.27–1.45)	<.001
Year of diagnosis	1.00 (0.95–1.05)	.97
Residence in NA	0.80 (0.73–0.88)	<.001
Random effects		
*s* ^2^	0.89	
*t*_00_ Group RD	0.95	
ICC	0.22	
*N* _Group RD_	24	
Marginal *R*^2^/Conditional *R*^2^	0.02/0.23	

**Figure 2.**
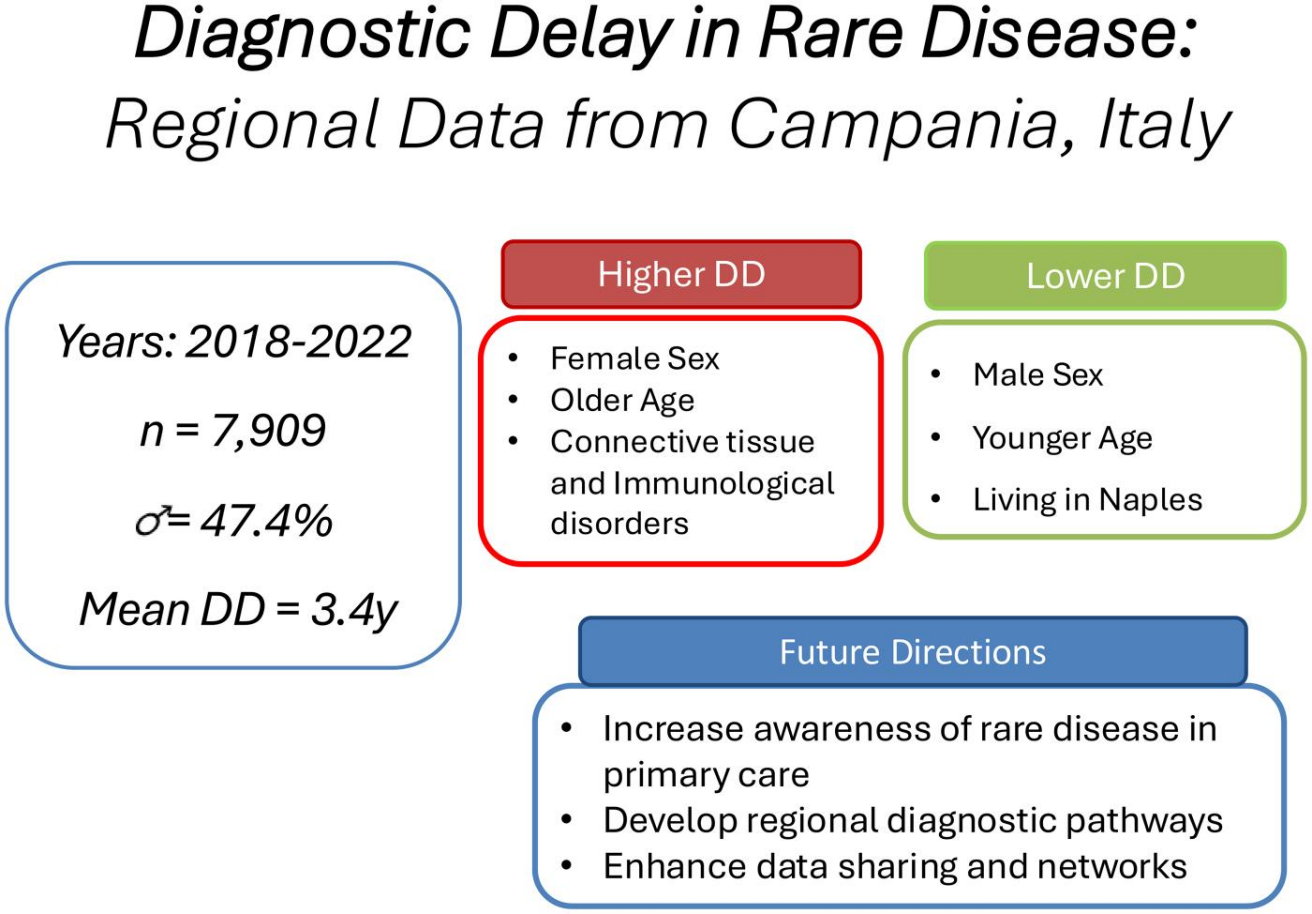
Central illustration summarizing study findings on diagnostic delay in rare diseases in Campania, highlighting sex, age, and geographic disparities and potential public health strategies.

Variation in random intercepts for disease macro-groups showed that the risk of DD was significantly higher in immunological disorders (OR 2.08), in all subgroups of complex congenital malformations, especially in the complex and very rare subgroup (OR 4.53), in musculoskeletal system disorders (OR 2.23), and in digestive system diseases (OR 2.25). Hematological (OR 0.42), metabolic (OR 0.46), endocrine (OR 0.53), dermatological (OR 0.75), and respiratory diseases (OR 0.53) showed a lower risk of significant DD (Supplementary Fig. S2). Study results are summarised in Figure 2.

## Discussion

### Total diagnostic time and international trends

This study reveals that almost half of patients diagnosed with a RD in Campania, Italy, experience some level of DD, with an average diagnosis time of 3.4 years from symptom onset. Overall, DD in RD has decreased over time.

While a 3.4-year delay in RD diagnosis does not meet the one-year goal set by the IRDiRC, it is shorter than the average DD reported by Faye and colleagues in a Europe-wide social media survey, and lower than the DD experienced by RD patients in Spain [4, 5]. The relatively better diagnostic performance and reduction in DD over time in Campania may be partially due to the presence of a dedicated RDRCC that oversees RD patient care and facilitates RD networks among specialists and patient associations [6, 7].

### Gender and DD

Our findings align with previous studies in Spain and the Europe-wide survey, showing that women are consistently diagnosed later than men [4, 5]. The delay is not unique to RD; the gender health gap has been widely documented across various conditions, including acute coronary syndromes [8], cancer care [9], tuberculosis [10], and inflammatory bowel disease [11]. Women also experience significant delays also in gender-specific conditions like endometriosis [12].

Several factors may contribute to this gender-specific delay, including the perception of higher risk of certain diseases in men (especially when diseases are known to be more prevalent in men, such as cardiovascular disease), milder or atypical presenting symptoms, and potential sex-based biases in medical decision-making [13–15].

Additionally, women have traditionally been underrepresented in clinical trials, which affects the applicability of clinical evidence to female patients [16, 17]. To close the gender gap, public health systems should aim to provide equal care for women by increasing awareness of disease prevalence and presentation in women, enrolling more women in clinical trials, and actively addressing provider bias in interpreting women’s symptoms.

### Age and DD

Our data indicate that older age at diagnosis predicts DD in RD. This may be due to newborn screening programs, increased RD awareness among pediatricians, and the clinical severity of complex rare syndromes, which may lead to earlier diagnoses in pediatric patients. In Italy, Expanded Newborn Screening (ENS) was introduced by national law in 2016 and includes a broader range of metabolic and genetic conditions. Given our data collection window (2018–2022), ENS may have contributed to earlier diagnosis in some infants. Still, the timeframe may be too short to fully evaluate its long-term impact. Conversely, older patients are more likely to have confounding factors, such as comorbidities, contributing to delays. Additionally, it is intuitive that longer delays result in older age at diagnosis (see Fig. 2).

### Geography and DD

In our cohort, patients residing in Naples (the capital city of Campania) experienced significantly less DD. This finding may reflect easier access to reference centers located in the city teaching hospitals, which historically have been the first well-organized centers in Campania. Geographic disparities in healthcare, often described as the “postcode lottery effect”, have been observed in other areas of medicine and refer to variations in care based on location rather than health need [18, 19].

### DD in RD macro-groups

Greater DD was observed in connective tissue and musculoskeletal disorders, immunological disorders, and congenital malformations and syndromes.

For connective tissue and musculoskeletal disorders, patients often experience significant delays before receiving diagnoses for conditions like polymyositis, mixed connective tissue disease, and systemic sclerosis. For example, systemic sclerosis has an average time to diagnosis of 6.3 years [20]. Our findings in immunological disorders also align with existing data on rare immunological conditions; for instance, familial Mediterranean fever often takes over 10 years to diagnose due to nonspecific symptoms, social factors, and gender differences [21].

Among all macro-groups, congenital malformations (including complex congenital and inherited syndromes) had the highest risk of DD. While these conditions typically present with early and severe symptoms, determining their exact causes can be challenging due to their rarity, which often requires a multidisciplinary approach.

### Public health implications

Despite the progress, a fifth of RD patients in Campania still experience a DD of over 4 years, and this poses a significant public health challenge. Potential improvements include increasing RD awareness and improve RD clinical pathways within the regional/national network. RD awareness should be implemented throughout all the medical school teaching and as postgraduate offer for different specialities, and with master degrees and doctorate offers. This should include specific attention on gender differences in the diagnosis and, potentially, management of rare disease in females.

In Campania region, a second regional public health plan for RD was recently approved. The current plan focuses on the reorganization of regional RD networks (with over 400 clinical experts working across 12 hospitals, six of which are part of the European networks for rare disease (details available in Supplementary Table S1)) simplifying and standardizing clinical pathways to guide diagnosis and management of each specific RD (PDTA, percorsi diagnostici terapeutici assistenziali), facilitating access to reference centers, and strengthening links between district hospitals and tertiary centers [19]. Moreover, education activities such as a master’s degree in RD (https://www.scienzemedichetraslazionali.unicampania.it/didattica/master-corsi-di-perfezionamento-e-di-alta-formazione-summer-winter-school/10-didattica/225-master-di-ii-livello-malattie-rare-master-in-clincal-science-on-rare-diseases), and tailored for general practitioners and pediatricians, have been implemented and prioritized inside the rare disease regional plan [22].

### Limitations

This study has several limitations. First, it is a retrospective analysis based on public registry data. Second, the study period (2018–2022) may have been impacted by the COVID-19 pandemic, which could have contributed to increased DD in some cases [23, 24].

## Conclusions

This is the first study able to quantify DD in RD in Italy. Our data support the notion that RD patients in Campania experience significant delays, though these delays are somewhat less than those reported in other studies. We found that DD has decreased over time. Still, the main determinants remain female gender, older age, connective tissue and immunological diseases, complex malformations, and geographic disparities, which may reflect unequal access to healthcare, requiring specific and measurable public health actions.

## Supplementary Material

ckaf088_Supplementary_Data

## Data Availability

The data underlying this article will be shared on reasonable request to the corresponding author. Key points Our study assessed time to diagnosis of rare diseases (RD) in Campania and whether there are clinical and demographic determinants of diagnostic delay (DD). Nearly half of patients with RD experienced DD. The main determinants of DD were female gender, older age at diagnosis. Immunological, connective tissue, digestive, genitourinary system diseases, and congenital malformations showed more DD than other disorders. The main determinants remain female, gender, older age, connective tissue and immunological diseases, complex malformations, and geographic disparities, which may reflect unequal access to healthcare, requiring specific and measurable public health actions.
