# Supplementary material for: Diagnostic delay in rare diseases in the Campania region: addressing ageing, gender disparities, and the “postcode lottery effect” to reduce the patient odyssey
Source: Eur J Public Health. 2025 Jul 4;35(5):862–6. doi: 10.1093/eurpub/ckaf088 (PMC12529253; doi:10.1093/eurpub/ckaf088)
Supplement: ckaf088_Supplementary_Data [file ckaf088_supplementary_data.zip › ckaf088_Supplementary_Data/ejph-2024-11-om-0836-File006.docx]

Supplementary Table 1

| Ospedale Antonio Cardarelli | ERN EuroBlood Net  Endo-ERN |
| --- | --- |
| AOU Federico II | ERN EuroBloodNet  Endo-ERN  RARE-LIVER  MetabERN  RITA  ENR-RND  EURO-NMD  ERN-LUNG  ERN-ITHACA  ERN-EURACAN |
| AO Santobono-Pausilipon | ERKNet |
| AO Dei Colli | ERN-LUNG  ERN GUARD-HEART |
| Azienda Ospedaliera Unvirsitaria  Vanvitelli | ERN EuroBloodNet  Endo-ERN  ERKNet  MetabERN  Endo-ERN  ERN-EYE  EURO-NMD |
| Azienda Ospedaliera Universitaria  San Giovanni di Dio e Ruggi d’Aragona – Scuola Medica Salernitana | ERN-reCONNECT |

ERN, European Reference Networks; AO, Azienda Ospedaliera; RITA, Rare Immunodeficiency, Autoinflammatory and Autoimmune diseases; RND, Rare Neurological Diseases; NMD, neuromuscular disease; ITHACA, Rare Malformation Syndromes, Intellectuale and Other Neurodevelopmental Disorders; EURACAN, Expert care for Rare Adult Solid Cancers; ERKNet European Network on Rare Kidney Diseases
