# Supplementary figures and images for: Diagnostic delay in rare diseases in the Campania region: addressing ageing, gender disparities, and the “postcode lottery effect” to reduce the patient odyssey
Source: Eur J Public Health. 2025 Jul 4;35(5):862–6. doi: 10.1093/eurpub/ckaf088 (PMC12529253; doi:10.1093/eurpub/ckaf088)

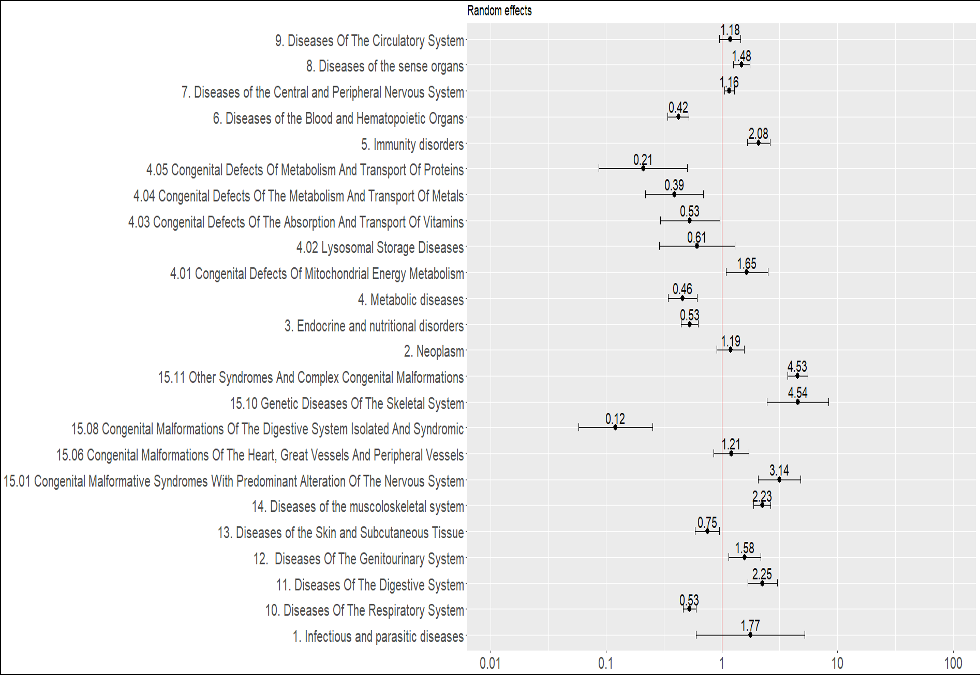

Supplement: ckaf088_Supplementary_Data [file ckaf088_supplementary_data.zip › ckaf088_Supplementary_Data/ejph-2024-11-om-0836-File004.tif]

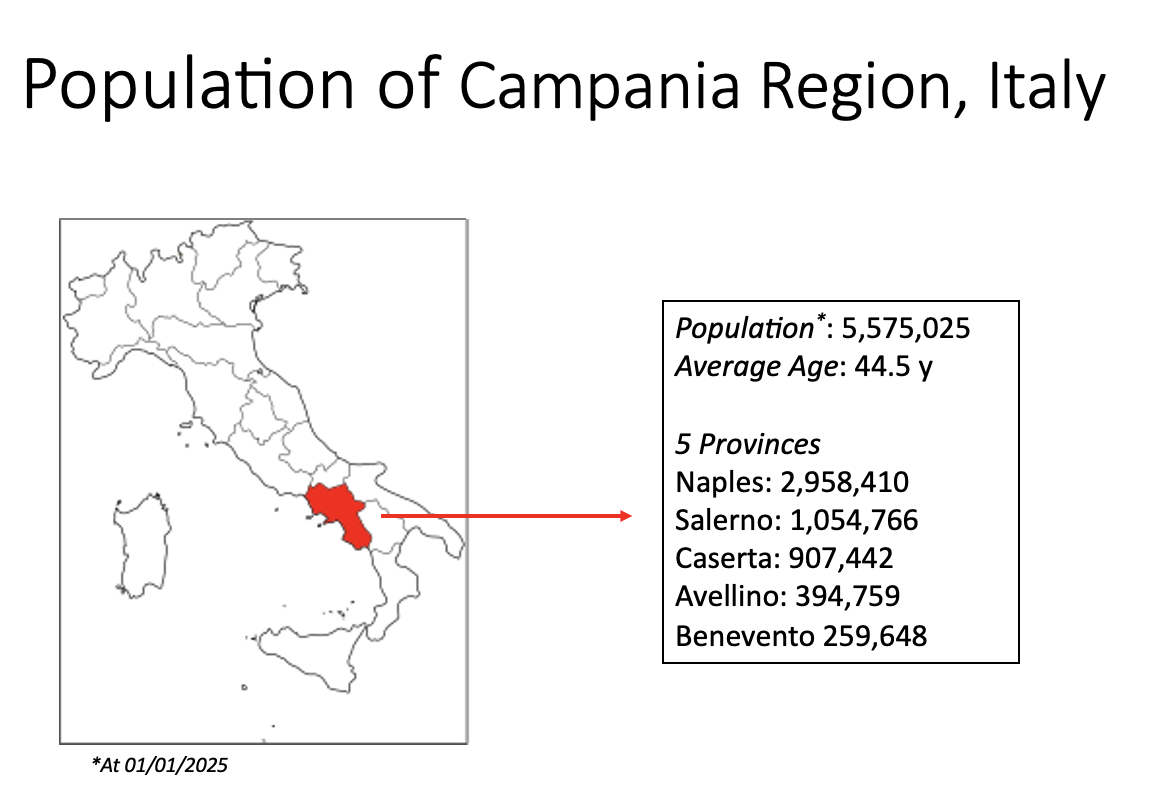

Supplement: ckaf088_Supplementary_Data [file ckaf088_supplementary_data.zip › ckaf088_Supplementary_Data/ejph-2024-11-om-0836-File005.tif]
